# MEOX2 Regulates the Growth and Survival of Glioblastoma Stem Cells by Modulating Genes of the Glycolytic Pathway and Response to Hypoxia

**DOI:** 10.3390/cancers14092304

**Published:** 2022-05-06

**Authors:** Carla Proserpio, Silvia Galardi, Maria Giovanna Desimio, Alessandro Michienzi, Margherita Doria, Antonella Minutolo, Claudia Matteucci, Silvia Anna Ciafrè

**Affiliations:** 1 Department of Biomedicine and Prevention, University of Rome Tor Vergata, 00133 Rome, Italy; carla.proserpio8@gmail.com (C.P.); silvia.galardi@uniroma2.it (S.G.); alessandro.michienzi@uniroma2.it (A.M.); 2 Research Unit of Primary Immunodeficiencies, Bambino Gesù Children’s Hospital, IRCCS, 00165 Rome, Italy; mariagiovanna.dsm@gmail.com (M.G.D.); doria@uniroma2.it (M.D.); 3 Department of Experimental Medicine, University of Rome Tor Vergata, 00133 Rome, Italy; antonella.minutolo@uniroma2.it (A.M.); matteucci@med.uniroma2.it (C.M.)

**Keywords:** MEOX2, glioblastoma stem cells, sphere formation, glycolytic enzymes

## Abstract

**Simple Summary:**

Glioblastoma is the most common incurable primary brain tumor in adults, typically leading to death within 15 months of diagnosis. Although there is an ongoing debate in the scientific community about the precise cellular origin of this tumor, glioblastoma stem cells (GSCs), which are able to self-renew, yield a full tumor mass, and determine chemo- and radio-resistance, are recognized to have a pivotal role. Our research aims to understand the role of the mesenchyme homeobox 2 (MEOX2) transcription factor in GSCs where it is strongly and specifically expressed. We have found that MEOX2 is indeed important for the survival of these cells. In fact, when we reduce its expression in two different GSC lines, they undergo a massive death accompanied by the inhibition of key genes of the glycolytic metabolism, the main source of energy for these cells. Our results reveal a novel function for MEOX2 in glioblastoma and suggest a mechanism through which GSCs may survive even in unfavorable conditions.

**Abstract:**

The most widely accepted hypothesis for the development of glioblastoma suggests that glioblastoma stem-like cells (GSCs) are crucially involved in tumor initiation and recurrence as well as in the occurrence of chemo- and radio-resistance. Mesenchyme homeobox 2 (MEOX2) is a transcription factor overexpressed in glioblastoma, whose expression is negatively correlated with patient survival. Starting from our observation that MEOX2 expression is strongly enhanced in six GSC lines, we performed shRNA-mediated knock-down experiments in two different GSC lines and found that MEOX2 depletion resulted in the inhibition of cell growth and sphere-forming ability and an increase in apoptotic cell death. By a deep transcriptome analysis, we identified a core group of genes modulated in response to MEOX2 knock-down. Among these genes, the repressed ones are largely enriched in genes involved in the hypoxic response and glycolytic pathway, two strictly related pathways that contribute to the resistance of high-grade gliomas to therapies. An in silico study of the regulatory regions of genes differentially expressed by MEOX2 knock-down revealed that they mainly consisted of GC-rich regions enriched for Sp1 and Klf4 binding motifs, two main regulators of metabolism in glioblastoma. Our results show, for the first time, the involvement of MEOX2 in the regulation of genes of GSC metabolism, which is essential for the survival and growth of these cells.

## 1. Introduction

Glioblastoma (GBM) is a grade 4 glioma, the highest among these brain tumors, inevitably fatal, and characterized by a very short survival after diagnosis (i.e., 20.9 months, notwithstanding the latest therapeutic options) [1,2,3]. A widely accepted classification of glioblastoma based on gene expression patterns was proposed in 2010 by Verhaak et al., distinguishing four subtypes, namely classical (CL), mesenchymal (MES), proneural (PN), and neural (NL) tumors [4]; more recently the same group revised this classification, removing the NL subtype, and highlighting the plasticity of all subtypes, able to switch from one to another [5]. A recent wide-range study has proposed that most glioblastomas may be subtyped in two main groups, type I and type II, reflecting two different cell-of-origin lineages characterized by either high EGFR and SOX9 or high ERBB3 and SOX10 expression, respectively [6], that has important therapeutic implications.

Extensive research on the initiation of this tumor has led to the general agreement that glioblastoma likely arises from stem-like cells though their origin is still highly debated: a hypothesis of normal stem or progenitor cells undergoing specific genetic aberrations is opposed by an alternative model of committed cells de-differentiation into stem-like cells [7]. Whatever is their origin, these glioblastoma stem like-cells (GSCs) are deemed responsible for the high chemo and radio-resistance of this tumor and for its recurrence, in most cases, the ultimate cause of patient death [8,9]. GSCs reflect the huge diversity typical of glioblastoma and represent an ideal model to study the tumor’s molecular basis. In the last decade, many efforts have been made to uncover gene expression signatures that are pivotal for GSC functions, and interesting results are now available about the transcriptome and the proteome of glioblastoma and its initiating cells [10,11,12,13,14,15,16,17].

Transcription factors containing homeodomains work as developmental regulators and are strongly implicated in tumors, including glioblastoma [18,19,20,21]. While many of these factors are encoded by clustered *HOX* genes, MEOX2 is encoded by an isolated gene located on human chromosome 7p21.2 [22]. A few papers have been published to date on the role of MEOX2 in glioblastoma, overall showing its overexpression in all types of glioma vs. healthy brain, a negative correlation of MEOX2 expression with survival, and also an enrichment of its expression in GBM patients who do not respond to radiotherapy [23,24,25]. MEOX2 expression was described as part of the molecular signature of the CL subtype [4] and was included in a 17-gene high-risk signature correlating with overall survival in mesenchymal glioblastomas [26]. One recent paper showed that GSCs could be classified into two groups based on distinct enhancer profiles and on the differential activity of specific developmental transcription factors, among which MEOX2 characterizes group 1 with PN and CL features [27]. These findings are important because, not only do they proposing a chromatin-based landscape definition of glioblastomas, they identify core transcription factors required for the growth of glioma cells of the two different subgroups and possibly represent druggable targets. Even more recently, a paper described the nuclear localization of MEOX2 in both GSCs and in glioblastoma tissues, suggesting its potential involvement in GSC phenotype and adhesion properties [28].

However, the investigation of the role of MEOX2 in glioblastoma stem cells is in its infancy, and we still need to understand the molecular mechanisms linking MEOX2 with the onset and aggressiveness of glioblastoma. Herein, we show that MEOX2 is strongly overexpressed in GSCs compared to stable cell lines, and we demonstrate that MEOX2 function is important for specific features of glioblastoma stem cells, in particular their survival and their ability to form spheres.

## 2. Materials and Methods

### 2.1. Cell Culture

Two human glioblastoma multiforme cell lines, U87 and T98G, and patient-derived glioblastoma stem cell (GSC) lines (a generous gift from I.R.C.C.S. Foundation, Neurological Institutes Carlo Besta, Milan, Italy), were used as experimental models. All the GSCs were derived from surgical samples of consecutive primary GBMs, which were obtained at the Fondazione IRCCS Istituto Neurologico C. Besta, according to a protocol approved by the institutional Ethical Committee, and were previously described [29,30]. For all experiments, GSCs were grown in vitro for less than 10 passages. As described in De Bacco et al., 2021 [29], and with reference to Wang Q. et al. 2017 classification [5], BT373, BT462, and BT273 were classified as Proneural, BT517, BT379, and BT417 as Classical; based on Wang Z. et al. 2020 subtyping [6], BT373 were group II, BT462 and BT417 group I, BT517, BT273, and BT379 group non I-II.

Normal human astrocytes isolated from human cerebral cortex (ScienCell #1800) were cultured in Astrocyte Medium (ScienCell #1801). Total human brain RNA from 3 healthy donors was purchased from Clontech (# 636530).

U87 and T98G were cultured as adherent cells in DMEM (Corning, Corning, NY, USA) supplemented with 10% FBS (Aurogene, Roma, Italy), 1% penicillin/streptomycin (Corning), and 1% L-glutamine (Aurogene). GSCs were cultured as floating spheres in DMEM/F-12 (1:1) (1X) + GlutaMAX (Gibco, Waltham, MA, USA) containing 1% penicillin/streptomycin (Corning), 1% L-glutamine (Aurogene), 2% B27 (Gibco), 0.1% heparin (Sigma-Aldrich, Waltham, MA, USA), 0.002% bFGF (PeproTech, Suzhou, China), and 0.002% EGF (PeproTech) at 37 °C in a humidified 5% CO_2_ incubator.

### 2.2. Lentiviral Vectors and Infections

To deplete MEOX2 endogenous expression, BT273 and BT379 cells were transduced with lentiviral particles containing the pLKO.1 vector (Merck, Kenilworth, NJ, USA) expressing shRNAs directed against MEOX2, or the SHC001 negative control vector (Merck). The MEOX2-targeting shRNA sequences were TRCN0000018253 (Merck), which we renamed shRNA53 for simplicity, and TRCN0000427218 (Merck), renamed shRNA18 for simplicity. While shRNA53 is directed against MEOX2 3′UTR, shRNA 18 targets the coding sequence of MEOX2. The sequences of the shRNA inserts were the following:

shRNA53: 5′-CCGGGCATTCATATTAGCTGATGAACTCGAGTTCATCAGCTAATATGAATGCTTTTT-3′
shRNA18: 5′-CCGGCATCAGAGCTGTCGGGAATTGCTCGAGCAATTCCCGACAGCTCTGATGTTTTTTG-3′

For the production of lentiviral particles, lentiviral vectors were co-transfected with the packaging vectors pLP1, pLP2, and VSV-g (Invitrogen, Waltham, MA, USA) into HEK293T cells using LipofectamineTM 3000 (Life Technologies) according to the manufacturer’s instructions. For transduction, 1 mL of concentrated viral supernatant and 1 µL of polybrene^®^ (8 µg/µL, Sigma) were added to the cell pellet. The transduced cells were then centrifuged at 2000× *g* for 1 h at room temperature. Finally, the viral supernatant was removed, and washed with DMEM/F-12 (1:1) (1X) + GlutaMAX (Dulbecco’s Modified Eagle’s Medium F-12 Nutrient Mixture, Gibco) was performed. The transduced cells were then grown in 6 mL of DMEM/F-12 culture medium, and after 48 h, 0.75 µg/mL of puromycin (Sigma) was added for the selection of stably transduced cells.

### 2.3. RNA Extraction and qRT-PCR

The total RNA was prepared from the transfected cells using TRIzol^®^Reagent (Invitrogen) according to the manufacturer’s instructions or from the GSCs using the Direct-zol™ RNA MiniPrep kit (Zymo Research, Irvine, CA, USA). RNA was quantified using a NanoDrop ND 1000 Spectrophotometer (Thermo Scientific), and 1 µg of RNA was treated with DNase I RNase-free (Biolabs, San Francisco, CA, USA). Then it was reverse transcribed using M-MLV RT (Invitrogen) following the manufacturer’s instructions. The resulting cDNA (25 ng) was used for the Real-time qPCR analysis using the Luna^®^ Universal qPCR Master Mix (New England Biolabs, NEB, Ipswich, MA, USA) on a StepOnePlus instrument (Applied Biosystem, Waltham, MA, USA) according to the protocol provided by the manufacturer.

The primers used were the following:

- ACTIN Forward: 5′-GCACTCTTCCAGCCTTCC-3′
- ACTIN Reverse: 5′-TGTCCACGTCACACTTCATG-3′
- MEOX2 Forward: 5′-GCAAGAGGAAAAGCGACAG-3′
- MEOX2 Reverse: 5′-CTTTCCTGGGTTTGCTGTTG-3′
- PPP2CA Forward: 5′-AGGAGCTGGTTACACCTTTG-3′
- PPP2CA Reverse: 5′-GCACCAGTTATATCCCTCCATC-3′

### 2.4. Protein Extraction and Western Blot Analysis

Cells were centrifuged at 1200 rpm for 10 min at 4 °C. The cellular pellet was lysed in NP40 Buffer (150 mM NaCl, 50 mM Tris-HCl pH 8.0, 0.5% NP40, 10% glycerol) plus protease inhibitor cocktail 50X (Promega, Milan, Italy), incubated on ice for 30 min and centrifuged at 13,000 rpm for 30 min at 4 °C. The supernatant was then collected into a new tube, and protein concentration was determined by the Bradford method. Equivalent amounts of protein extract were separated by electrophoresis on 10% or 12% SDS-PAGE gels and blotted onto nitrocellulose. The membranes were blocked with 5% non-fat dry milk and 0.1% Tween-20 in Phosphate-buffered saline and then incubated with antibodies followed by the appropriate horseradish peroxidase-conjugated secondary antibodies (1: 8000, Promega, Milan, Italy). After three washes in PBS Tween-20 0.1%, the signal was developed with the ECL system (Santa Cruz Biotechnology, INC., Dallas, TX, USA) according to the manufacturer’s protocol. The primary antibodies employed for protein detection were: Anti-alpha-Tubulin (Sigma-Aldrich, T8203, 1:5000), Anti-MEOX2 (Sigma, HPA053793, 1:2250), Anti-cleaved Caspase-3 (Cell Signaling Technology, Danvers, MA, USA, BK9664, 1:1000), anti-Caspase-3 (GeneTex, Irvine, CA, USA, GTX110543, 1:1000), anti-HK2 (abcam, ab209847, 1:1000), anti-AldoC (GeneTex, GTX102284, 1:1000), anti-PFKFB4 (GeneTex, GTX107755, 1:1000). Original Western Blot figures shown in File S1. 

### 2.5. Sphere Formation Analysis

BT273 and BT379 were plated in triplicate in a 12-well plate (3000 cells/well) in DMEM/F-12 (1:1) (1X) + GlutaMAX (Gibco). After seven days, the number and size of the neurospheres were evaluated by acquiring photos of the wells (Nikon ECLIPSE TS100). We scored as actively growing spheres those with a diameter ≥ 50 µm. Three experiments were performed in triplicate.

### 2.6. Growth Assay on Geltrex^®^ Coated Plates

Then, 0.3 mL Geltrex^®^ Ready-To-Use matrix (Gibco) was used for coating the wells of a 24-well plate, which was subsequently incubated for one hour at 37 °C to allow gelling of the matrix. At the time of use, the liquid layer above the Geltrex^®^ coating was aspirated off, and the GSCs were transduced with the viral supernatant pLKO.1-puro-ctrl, or pLKO.1-puro-shRNA18 or pLKO.1-puro-shRNA53, in pre-equilibrated DMEM/F-12 (1:1) (1X) + GlutaMAX (Gibco) and were immediately plated (20,000 cells/well) in triplicate. Cells were detached with 50 µL of Trypsin-EDTA 1X in PBS (EuroClone, Pero, Italy) and 50 µL of DPBS 1 × (Dulbecco’s Phosphate-Buffered Saline, Corning) were added to the detached cells. Then, 50 µL of this cell suspension were mixed with 50 μL of trypan blue stain 0.4% (Gibco) to be counted at the different time points using a Neubauer chamber (Marienfeld). Live cells were counted using a Nikon ECLIPSE TS100 microscope.

### 2.7. Cytofluorimetric Analysis of Apoptosis

Apoptosis was assessed by flow cytometry analysis using a CytoFLEX (Beckman Coulter, Boulevard Brea, CA, USA) on isolated nuclei stained with Propidium Iodide (PI) (Merck KGaA, Darmstadt, Germany) using a method that distinguishes nuclei from apoptotic, necrotic, or viable cells, as previously described [31]. Early apoptotic events were detected through double-staining of the cells with fluorescent annexin-V and with a 7-amino actinomycin D (7-AAD) solution. For this purpose, the “Annexin V-FITC Kit 7-AAD (IM3614, Beckman Coulter) was used according to the manufacturer’s instructions. Briefly, 5 × 10^5^ cells were incubated for 15 min with annexin-V-fluorescein isothiocyanate and then washed in annexin buffer. Cells were then stained with 7-AAD and analyzed immediately after staining by flow cytometry analysis. Data acquisition and analyses were performed using the CytExpert 2.0 (Beckman Coulter, Carlsbad, CA, USA) using a minimum of 150,000 events for each sample.

### 2.8. RNA-Seq Analysis of BT273 and BT379 GSCs

The total RNA was extracted from cells using the Direct-zol RNA MiniPrep Kit (Zymo Research). The RNA library preparation, sequencing reaction, and bioinformatics analysis were conducted at GENEWIZ Germany GmbH (Leipzig, Germany). The extracted RNA samples were quantified using a Qubit 2.0 Fluorometer (Life Technologies, Carlsbad, CA, USA), and the RNA integrity was checked using an Agilent Fragment Analyzer (Agilent Technologies, Palo Alto, CA, USA). The RNA sequencing libraries were prepared using the NEBNext Ultra II RNA Library Prep Kit for Illumina using the manufacturer’s instructions (New England Biolabs, Ipswich, MA, USA). Briefly, mRNAs were initially enriched with Oligo d(T) beads. The enriched mRNAs were fragmented for 15 min at 94 °C. First-strand and second-strand cDNA were subsequently synthesized. cDNA fragments were end-repaired and adenylated at 3’ends, and universal adapters were ligated to cDNA fragments, followed by index addition and library enrichment by PCR with limited cycles. The sequencing libraries were validated on the Agilent Fragment Analyzer (Agilent Technologies, Palo Alto, CA, USA) and quantified by using the Qubit 2.0 Fluorometer (ThermoFisher Scientific, Waltham, MA, USA). The sequencing libraries were multiplexed and clustered on the flowcell. After clustering, the flowcell was loaded onto the Illumina NovaSeq 6000 instrument according to the manufacturer’s instructions. The samples were sequenced using a 2 × 150 Pair-End (PE) configuration. The raw sequence data (.bcl files) generated from Illumina NovaSeq were converted into fastq files and de-multiplexed using the Illumina bcl2fastq program version 2.20. One mismatch was allowed for index sequence identification. After investigating the quality of the raw data, sequence reads were trimmed to remove possible adapter sequences and nucleotides with poor quality using Trimmomatic v.0.36. The trimmed reads were mapped to the Homo sapiens GRCh38 reference genome available on ENSEMBL using the STAR aligner v.2.5.2b. BAM files were generated as a result of this step. Unique gene hit counts were calculated by using feature Counts from the Subread package v.1.5.2. Only unique reads that fell within exon regions were counted. After the extraction of gene hit counts, the gene hit counts table was used for downstream differential expression analysis. Using DESeq2, a comparison of the gene expression between the groups of samples was performed. The Wald test was used to generate *p* values and Log2 fold changes. Genes with adjusted *p* values < 0.05 and absolute log2 fold changes >0.7 were identified as the differentially expressed genes for each comparison. The RNAseq data (fastq files) were deposited in the GEO database under the accession number GSE196141. Gene ontology analysis was performed on the statistically significant set of genes by implementing the GeneSCF software. The GO list was used to cluster the set of genes based on their biological process and to determine their statistical significance.

### 2.9. Transcription Factors (TF) Binding Sites Enrichment Analysis

TF binding sites over-representation analysis was performed using oPOSSUM (v.3.0, Single Site Analysis tool) [32]. Differentially regulated genes were used as targets, and all genes measured by our RNA-seq were used as a background (conservation cutoff: 0.6; matrix score threshold: 85%; upstream/downstream region: 5kb/5kb; JASPAR CORE Profiles: All vertebrate profiles).

## 3. Results

### 3.1. MEOX2 Depletion Inhibits the Sphere-Forming Ability and Induces Apoptosis in Glioblastoma Stem Cells

**Figure 1:**
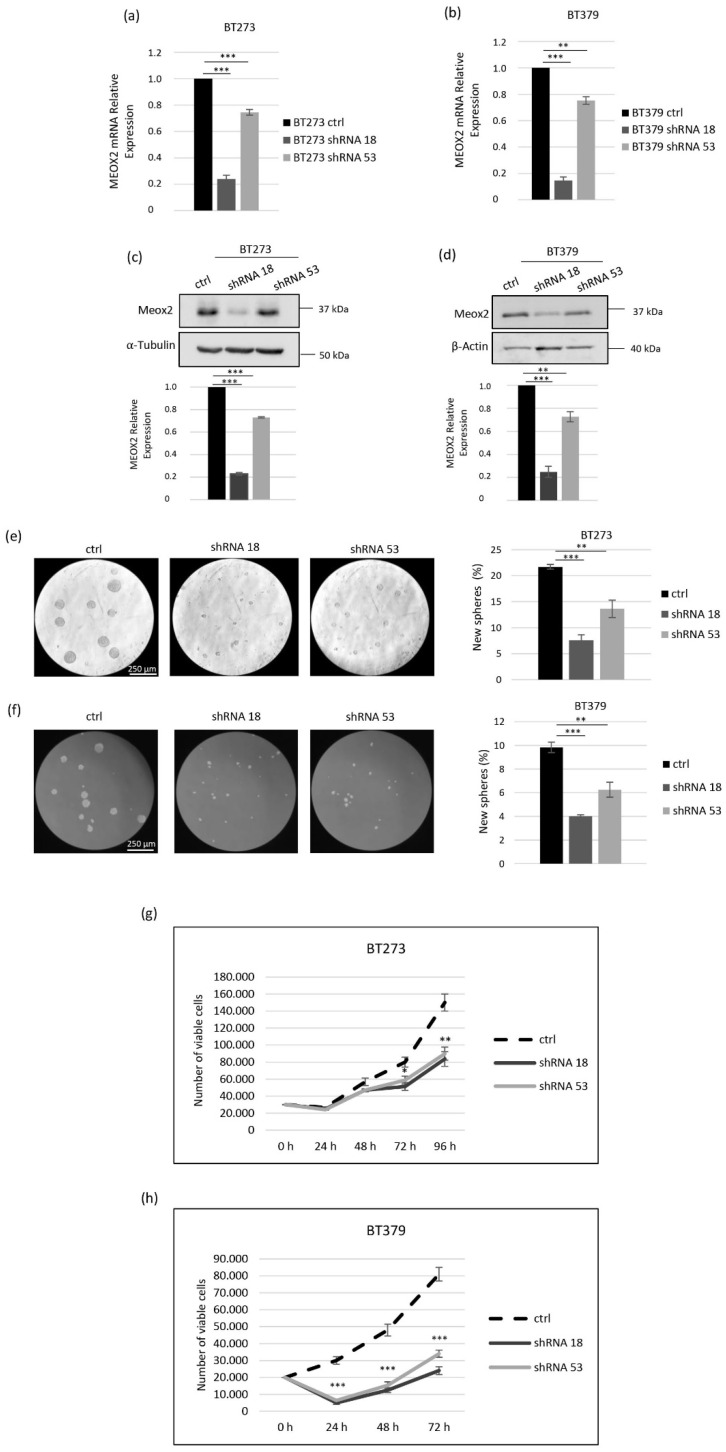
MEOX2 depletion inhibits the sphere-forming and the growth ability of glioblastoma stem cells BT273 and BT379. (**a**,**b**) MEOX2 qRT-PCR analysis of BT273 (**a**) or BT379 (**b**) cells transduced with shRNA18 or shRNA53 or ctrl lentiviral constructs. The values were reported in relation to cells transduced with ctrl vector set as = 1 and normalized to PPP2CA mRNA expression (*n* = 3; mean ± SD). (**c**,**d**) MEOX2 Western blot analysis of BT273 (**c**) or BT379 (**d**) cells transduced with shRNA18 or shRNA53 or ctrl lentiviral constructs. For BT273 and BT379, α-tubulin and β-actin were used as the internal loading controls, respectively. Representative images are shown. The bottom histograms show the quantification of MEOX2 in relation to α-tubulin and β-actin. (*n* = 3; mean ± SD). (**e**,**f**) Sphere-forming assay of BT273 (**e**) and BT379 (**f**) cells transduced with shRNA18 or shRNA53 or ctrl lentiviral constructs. Histograms show the percentage of cells capable of re-forming a neurosphere seven days after dissociation (*n* = 3; mean ± SD). Representative micrographs are shown. (**g**,**h**) Growth curves of BT273 (**g**) and BT379 (**h**) cells transduced with shRNA18 or shRNA53 or ctrl lentiviral constructs. (*n* = 3; mean ± SD). Differences between two groups were assessed using unpaired Student’s *t*-test (two-tailed). Significance was defined as * *p* < 0.05; ** *p* < 0.01; *** *p* < 0.001, statistical difference compared to the control.

We analyzed the MEOX2 expression in six patient-derived GSCs populations and found that it was strongly expressed in all cell lines, whereas it was absent or barely expressed in all other non-stem samples used as controls, either healthy brain tissue and astrocytes or stable glioblastoma cell lines (Figure S1). With the aim of unravelling if such a strong overexpression of MEOX2 plays a functional role in GSCs, we depleted it by shRNAs delivered via lentiviral vectors. We initially assayed two shRNAs, namely shRNA18 and shRNA53, in two different GSC lines, BT379 and BT273, chosen as they belong to two different subtypes, i.e., the CL and the PN ones, respectively [30], and express comparable high levels of MEOX2 mRNA (Figure S1). In both BT379 and BT273 lines, shRNA18 reduced MEOX2 more strongly than shRNA53 at the mRNA and protein levels (Figure 1a–d). Given that self-renewal is a key aspect of cancer stem cells, we tested if MEOX2 depletion affected BT273 and BT379’s ability to reassemble into new spheres after dissociation to single cells. As shown in Figure 1e,f, in both cell lines, the strong reduction in MEOX2 expression obtained by shRNA18 was associated with a drastic decrease in the sphere-forming ability. This GSC activity, conversely, was only slightly affected by shRNA53, which was unable to effectively knock down MEOX2. Of note, not only was the number of spheres reduced upon MEOX2 knock-down, but also their size, in particular in BT379 cells. This may indicate that MEOX2 depletion affects not only the bare ability of GSCs to form new spheres but also their growth and/or viability.

Then, we plated both BT379 and BT273 cells transduced with either shRNA18 or shRNA53 onto Geltrex coated plates, with the aim of counting the number of living cells while avoiding the formation of spheres. The results of such assays, depicted in Figure 1g,h, clearly show that MEOX2 knock-down had an impact on the growth ability of both cell lines, even if with some differences. In BT273 cells, MEOX2 depletion resulted in a clear reduction in viability, which was significant at 72 and 96 h from plating (Figure 1g). For BT379 cells, a reduction was evident in knocked down cells at 24 h after plating and slowly recovered at later time points (Figure 1h), suggesting that those cells that succeeded in attaching and then regained a growth rate similar to control cells. Thus, the depletion of MEOX2 in BT379 cells deeply affects their ability to survive and, consequently, to attach to plates. The reduced viability/increased death of MEOX2-depleted cells might account for the reduced size of the spheres observed in the sphere-formation experiments (Figure 1e,f).

**Figure 2:**
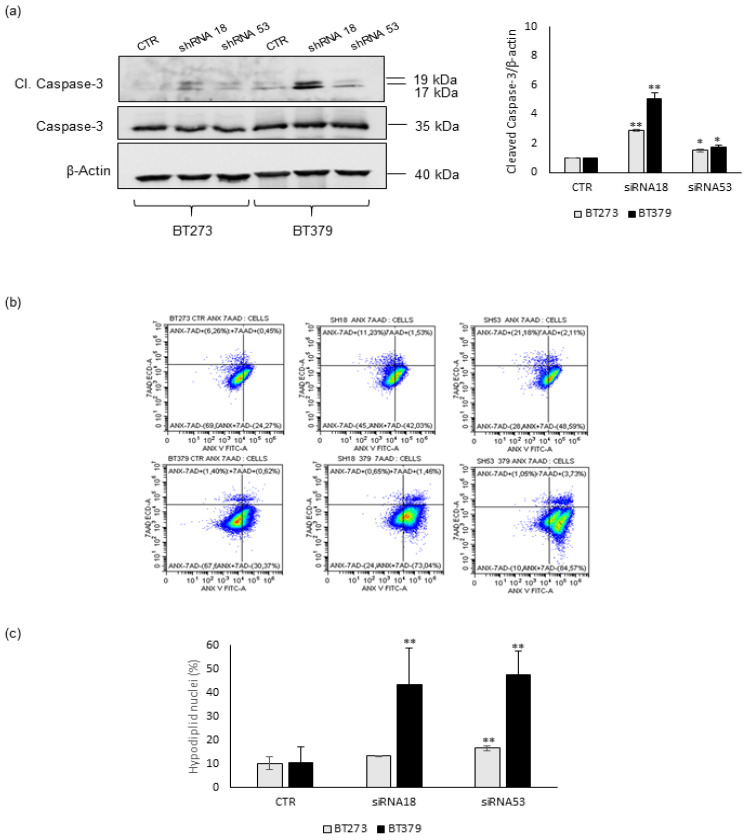
MEOX2 depletion induces the apoptosis of glioblastoma stem cells BT273 and BT379. (**a**). Cleaved Caspase-3 Western blot analysis of BT273 and BT379 cells transduced with shRNA18 or shRNA53 or ctrl lentiviral constructs. Total Caspase-3 is shown. β-actin was used as the internal loading control. (*n* = 3; mean ± SD). One representative image is shown. The histograms on the right show the quantification of Cleaved Caspase-3 in relation to β-actin. (Differences between two groups were assessed using unpaired Student’s *t*-test (two-tailed). Significance was defined as * *p* < 0.05; ** *p* < 0.01, statistical difference compared to the control). (**b**). One representative dot plot of the percentage of Annexin V/7AAD positive and negative cells analysed by Flow cytometry analysis; the mean values ± s.d. of three independent experiments performed were reported in Table 1. (**c**). FACS analysis based on Propidium Iodide staining of hypodiploid nuclei fraction (%) in either BT273 or BT379 GSCs transduced with shRNA18 or shRNA53. Data are presented as mean values ± s.d. Differences between two groups were assessed using unpaired Student’s *t*-test (two-tailed). Significance was defined as ** *p* ≤ 0.01.

To check if the reduction in viability observed upon MEOX2 depletion may be due to the induction of the apoptotic pathway, we assayed Caspase-3 cleavage in BT273 and BT379 cells transduced with either shRNA18 or shRNA53. In both cell lines, MEOX2 knock-down mediated by both shRNAs induced an increase in Caspase 3 processing, even if this was to different extents (Figure 2a). In addition, Caspase 3 activation was much stronger in BT379 than in BT273 cells, confirming our data on the stronger reduction in the viability of MEOX2-depleted BT379 cells.

In agreement with these data, when we assessed the extent of apoptotic cell death by a propidium iodide-based flow cytometric assay, we found this was induced in both BT273 and BT379 upon MEOX2 depletion by shRNA18 or shRNA53 (Figure 2c), as also shown by flow cytometry analysis of 7-AAD (7-Aminoactinomycin D)-Annexin V staining (Figure 2b and Table 1).

### 3.2. MEOX2 Knock down Variably Modulates Gene Expression in Different GSC Lines, but Consistently Affects the Glycolytic Pathway and the Response to Hypoxia

Starting from our original finding of a great enrichment of MEOX2 expression in GSCs, we aimed to understand its role in this specific stem cell environment. Thus, we analyzed the transcriptome of BT273 and BT379 cells depleted of MEOX2. Our screening of differentially expressed genes (DEGs) upon MEOX2 knock-down was performed by setting a threshold of absolute log2 fold change ≥ 0.7 (adjusted *p*-value ≤ 0.05). This yielded very different numbers of DEGs in BT273 compared to BT379 cells, upon transduction with shRNA18: 171 genes were differentially expressed (among which 88 were induced and 83 were repressed) in BT273 cells (Table S1), whereas in BT379 cells, 1459 genes were affected (673 induced and 787 downregulated) (Table S1). In addition, we noticed that BT273 cells transduced with shRNA53 (less efficient than shRNA18 in knocking down MEOX2) showed differential expression of only 142 genes, among which 38 were upregulated and 104 downregulated (Table S1). This result indicated that in glioblastoma stem cells, MEOX2 expression perturbations affect gene expression in a very variable way, possibly depending on the context of the specific cell.

**Figure 3:**
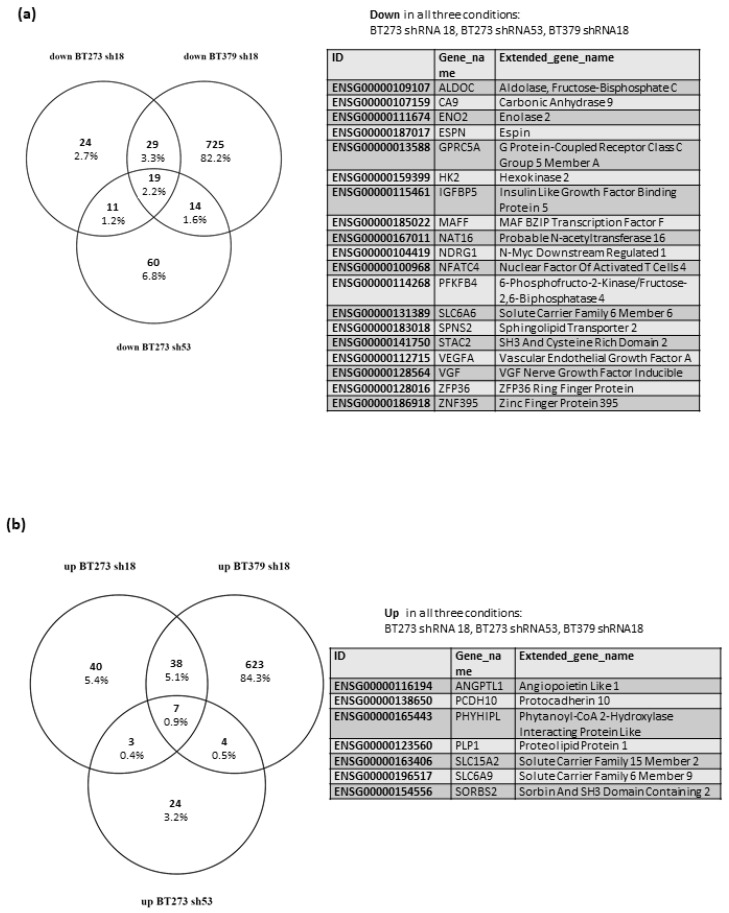
MEOX2 knock down in glioblastoma stem cells differentially modulates gene expression in different cells, but consistently affects some subsets. (**a**,**b**) Venn diagrams showing the numbers and percentages of DEGs (absolute log2 fold change ≥ 0.7, adjusted *p*-value ≤ 0.05) in BT273 or BT379 cells transduced with either shRNA18 or shRNA53, compared to the same cell types transduced with a negative control. Panel a shows the downregulated genes, and panel b the upregulated ones. Each diagram is flanked, on the left, by the list of genes consistently modulated in all three conditions, i.e., BT273 shRNA 18, BT273 shRNA53, BT379 shRNA18.

When we compared BT273 and BT379 cells transduced with shRNA18, we found 92 DEGs consistently modulated (48 downregulated and 45 upregulated), even if on such different backgrounds (Figure 3a,b and Table S1). Of note, 19 of the shared downregulated DEGs and seven of the upregulated ones were also (log2FC ≥ ±0.7 and adj *p*-value ≤ 0.05) modulated in BT273 cells transduced with shRNA53 (Figure 3a and Table S1), and an additional set of 24 (12 downregulated and 12 upregulated) were consistently modulated in BT273 cells-shRNA53, but either to a lesser extent or with a less significant *p*-value (Table S1). As a whole, this set of genes modulated in response to MEOX2 knock-down may represent a core collection of MEOX2 regulated genes in glioblastoma stem cells.

With the aim of inferring the main biological functions affected by the depletion of MEOX2 in GSCs, we performed a Gene Ontology analysis of DEGs (both down- and upregulated; absolute log2 fold change ≥ 0.7; adjusted *p*-value ≤ 0.05) (Table S2). The top Biological Process clearly enriched in BT273 cells transduced with either shRNA18 or shRNA53 was “Response to hypoxia”, while the much greater number of DEGs in BT379-shRNA18 resulted in a strong enrichment of GO-BPs related to mitosis and mitotic spindle organization. The term “negative regulation of mitotic metaphase/anaphase transition (GO:0045841)” was the most enriched one. This may well reflect our observations of the very reduced viability of MEOX2 knocked-down GSCs, in particular in BT379. Interestingly, terms such as “neuron development”, “neurogenesis”, and other related terms were also enriched in the BT379 cell line. We also noticed that, out of the 13 genes, all downregulated, included in the GO-BP term “response to hypoxia” enriched in BT273 knocked down for MEOX2, eight were also significantly repressed in BT379 shRNA18 (Table S2). This suggests that MEOX2 knock-down results in an impaired ability of both BT273 and BT379 cell lines to react to hypoxic, unfavorable environments.

We then submitted the 26 genes that were consistently modulated in all three comparisons (i.e., BT273 shRNA 18 vs. ctrl, BT273 shRNA53 vs. ctrl, BT379 shRNA18 vs. ctrl; see above) to Gene Ontology analysis and found a strong and significant enrichment of the Reactome Pathways “Glycolysis (R-HSA-70171)” and “Glucose metabolism (R-HSA-70326)” (Table S2). The four related DEGs (*PFKFB4*; *ENO2*; *ALDOC*; *HK2*) were downregulated in all three comparisons, and we also confirmed the repression of their protein products (Figure S2). This indicates that the DEGs shared in all three conditions mainly contribute to one shared biological function, which is the negative modulation of the glycolytic pathway.

### 3.3. Up- and Down-Regulated Genes in MEOX2-Depleted GSCs Differ for the GC Content in Regulatory Regions

**Figure 4:**
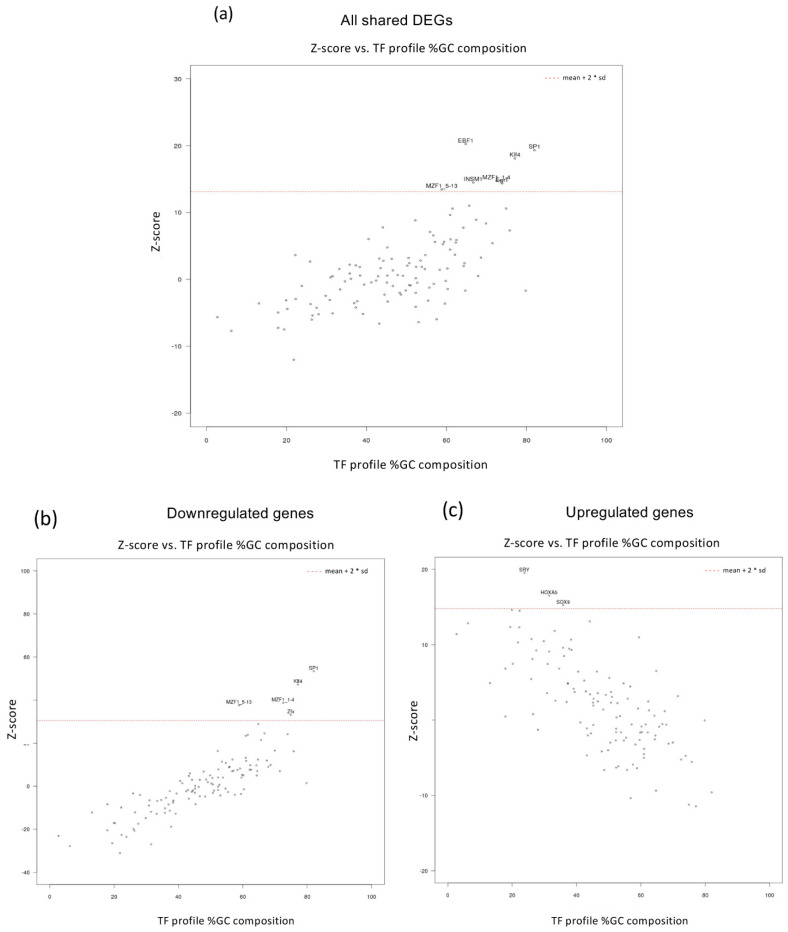
Enrichment analysis of transcription factor binding motifs in the regulatory regions of genes modulated by MEOX2 knock-down. (**a**) All DEGs (both down- and upregulated, in both BT273 and BT379 cells, by MEOX2 KD) were subjected to enrichment analysis of TF binding motifs using oPOSSUM-3 software. (**b**) Only downregulated DEGs analyzed as in (**a**). (**c**) Only upregulated DEGs analyzed as in (**a**). The names of the significantly enriched transcription factor binding motifs (Z score > mean + 2 SD) are shown.

MEOX2 is a transcription factor that plays a wide range of roles in cell development and in cancer, functioning as either a direct or indirect activator of its target genes [33,34,35,36]. In many cases, the mechanistic basis of its function in the different contexts has not been clarified yet. In addition, the consensus binding site for MEOX2, C/TAATTA, is an A/T rich sequence common to several other homeobox transcription factors. Thus, we used our differential gene expression data to study if the genes modulated by MEOX2 knock-down in GSCs share regulatory regions, which might be those recognized by MEOX2 or its interactors. By employing oPOSSUM-3, a system for determining the over-representation of transcription factor binding sites (TFBS) and TFBS families within a set of genes [32], we analyzed all DEGs (both down- and upregulated, in both BT273 and BT379 cells, by MEOX2 KD. The binding sites of the six transcription factors (TFs) SP1, Klf4, MZF1_1–4, EBF1, MZF1_5–13, and INSM1 were significantly enriched within regulatory regions (Z score > mean + 2 SD; Figure 4a) of genes affected by MEOX2 KD. We observed that most of the highest-ranking enriched TFs (Z score ≥ 10.00; Table S3) recognize DNA sequences with a high (>0.66) GC content. Interestingly, when we separately submitted downregulated or upregulated DEGs to oPOSSUM-3 analysis (defined as the “core down” or “core up” in Table S2), we observed that the top-ranking TF binding sites shared by downregulated genes were mainly represented by GC-rich regions, while the opposite was true (GC content < 0.33) when the analysis was performed on DEGs upregulated in response to MEOX2 KD (Figure 4b,c and Table S3). Sp1 and Klf4, both main regulators of metabolism in glioblastoma [37,38], were the highest-ranking transcription factors whose motifs were enriched in all DEGs, but in particular in the shared downregulated genes. We also obtained analogous results when we submitted DEGs from each of our cell types (BT273 or BT379), transduced with either shRNA (shRNA18 or shRNA53), compared to the controls (Figures S3 and S4 and Table S4). These results suggest that MEOX2 knock-down affects GSC biology by repressing a set of glycolysis-related genes, possibly interfering with their regulation by the transcription factors Sp1 and Klf4.

## 4. Discussion

Reflecting on the considerable heterogeneity of glioblastomas, the nature of GSCs is highly heterogeneous, which favors the capacity of these cells to elude therapies. Several attempts to classify glioblastomas and their initiating cells into functional and molecular groups have not achieved the goal of understanding the basis of such heterogeneity. In this complex frame, transcription factors surely play major roles in the maintenance of the varied phenotypes of cancer stem cells. MEOX2 is a homeodomain-containing transcription factor whose expression is extremely enriched in gliomas compared to all other types of tumors and was very recently found to be highly expressed in GSCs as well [28]. We were intrigued by the extreme overexpression of MEOX2 in all six GSC samples we analyzed, possibly indicating a role for this factor in glioblastoma initiating cells. MEOX2 RNA levels were all very high in our small cohort of GSCs, with no apparent distinction between subtypes, as defined by either Wang Q. et al., 2017 [5] or Wang Z. et al., 2020 [6]. As previously shown by us and others, MEOX2 is known to be overexpressed in glioblastomas compared to healthy brain cells [23,24,25,39], and our present data further highlighted that GSC lines express MEOX2 while established non-stem glioblastoma cell lines essentially lack its expression. The reported inhibition of MEOX2 expression by serum-containing media in vascular smooth muscle cells [40] is not sufficient to explain its absence in established cell lines, as we did not observe any induction of MEOX2 upon the growth of those cells in serum-free conditions (data not shown). This favors a stem-specific expression/role of MEOX2, at least in the context of glioblastoma.

As a readout of the stemness ability of GSCs, we measured their capacity to form new spheres and demonstrated that this function was inhibited after the stable knock-down of MEOX2. In addition, a clear reduction in viability characterized MEOX2-depleted cells that were prone to apoptotic death. We searched the transcriptome of the MEOX2 depleted cells to investigate the basis of the observed phenotypes, and, in line with the known great variability of glioblastoma stem cells, we found a large number of genes modulated by MEOX2 knock-down that were different in the two GSC lines we assayed. However, a “core set” of genes showed an overlapping differential expression as a consequence of MEOX2 repression. This group was impressively enriched in genes involved in the hypoxic response and in the glycolytic pathway, two strictly related pathways that contribute to the resistance of high-grade gliomas to therapies [41]. In fact, most solid tumors, and, in particular, glioblastomas, exploit the anaerobic glycolytic activity independent of oxygen supply in the so-called Warburg effect [42,43]. This phenomenon tightly applies to GSCs, whose metabolic shift is thought to be responsible for the high resistance to therapies of these cells upon induction of their self-renewal and invasive ability [44]. Notably, all the hypoxia- and glycolysis-related genes modulated in our GSCs after MEOX2 knock-down were negatively regulated. This suggests that MEOX2 may regulate the ability of GSCs to respond to the environment and to act as a metabolic shift. Indeed, when MEOX2 is repressed, these cells show high rates of apoptotic cell death.

The four metabolic genes whose repression is shared in both the BT273 and BT379 cell lines when MEOX2 is knocked down are all known to be induced by hypoxia and play a key role in the glycolytic pathway and have all been proposed as possible therapeutic targets for glioblastoma. *PFKFB4* is one of the four genes that encode 6-phosphofructo-2-kinase/fructose-2,6-biphosphatase, and its mRNA levels significantly differentiate *IDH1* wild-type primary glioblastomas from the secondary glioblastomas and from *IDH* mutant gliomas. Moreover, *PFKFB4* expression is an unfavorable prognostic marker in glioblastoma, as it inversely correlates with survival [45]. *HK2* encodes hexokinase 2, which mediates the first glycolytic step generating glucose-6-phosphate. *HK2* is a key driver of metabolic regulation, growth, and resistance to therapy in GBM, and its chemical inhibition resulted in the effective reduction in tumor growth in xenograft models of glioblastoma [46]. *ENO2* codes for Enolase2, which, together with *ENO1*, catalyzes the glycolytic production of phosphoenolpyruvate from 2-phosphoglycerate. Its repression strongly impairs tumor growth in glioblastoma xenografts [47]. Finally, *ALDOC* encodes for the C isozyme in the family of aldolases, which catalyzes the conversion of fructose 1,6-bisphosphatase to glyceraldehyde 3-phosphate and dihydroxyacetone phosphate (DHAP) during glycolysis, and is highly enriched in the brain as compared to any other healthy tissue, and in glioblastoma tissues [39], despite its mRNA levels being inversely correlated with glioma tumor grades (being higher in grade 2 and 3 gliomas, compared to grade 4 tumors) in one study [48].

Our prediction on the enrichment of binding sites for Klf4 and Sp1, and more generally for factors binding G-C rich motifs, in the regulatory regions of genes repressed by MEOX2 knock-down may indicate a mechanism through which MEOX2 works in GSCs, that is, acting as a modulator of the epigenetic state of G-C rich regulatory regions, also via the interference with Klf4 and Sp1 binding. More investigation is needed to unravel how MEOX2 could play this role, but a suggestive observation comes from ZNF395, one of the most commonly downregulated genes upon MEOX2 knock-down. ZNF395 is a transcription factor induced by hypoxia [49] and a mediator of the maximal hypoxic induction of proinflammatory cytokines in glioblastoma [50]. Its regulatory region largely overlaps with a CpG island, and ZNF365 itself is known to bind a CG-rich consensus sequence [51]. In the context of clear cell renal cell carcinoma, it was identified as a master regulator whose depletion results in tumor elimination, and it was shown that the epigenetic regulation of its transcription involves a “super-enhancer” [52]. These regions (defined as large enhancers located near genes encoding for master transcription factors of cell identity and disease) are relevant in glioblastoma and have been extensively studied in this tumor, together with their associated genes and core transcription factors that define the super-enhancers [53], particularly in GSCs, where they are pivotal to maintain GSC identity. Notably, MEOX2 was shown to be aberrantly activated in one of the two subgroups into which GSCs were classified based on super-enhancer chromatin states and was considered one of the master transcription factors of this subgroup [27].

It is thus intriguing to hypothesize that the results we obtained in GSCs depleted of MEOX2 are due to the role of MEOX2 as a master transcription factor involved in the modulation of the epigenetic state of super-enhancers in GSCs. Among these super-enhancers, MEOX2 might modulate those that drive the expression of ZNF395, and hypoxia-related genes, such as HK2 and VEGFA, that we found repressed by MEOX2 depletion.

A limitation of our current study is that our functional results were obtained in two GSC lines only. However, our study stemmed from our original observation of an extremely high enrichment of MEOX2 expression in all six of the GSC lines we assayed. In support of our experimental data, extensive MEOX2 expression is also reported in a separate, larger set of 48 GSC cultures, whose gene expression data can be downloaded from the human glioblastoma cell culture (HGCC) portal at www.hgcc.com, accessed on 27 January 2022 [54]. In the context of the well-known heterogeneity of glioblastoma and of its initiating cells, this points to the significance of MEOX2 enrichment in GSCs. While our manuscript was in preparation, a paper was published about MEOX2 in GSCs [28]. The results described therein are different from those we obtained in our GSC lines, both from the functional and molecular points of view. In fact, the authors showed a slight increase in cell viability (actually induced by only one siRNA targeting MEOX2) in three GSC lines, and the increased phosphorylation of ERK1/2 and AKT, upon MEOX2 depletion. This led the authors to claim that MEOX2 depletion positively affects the growth of GSCs through ERK/MAPK and PI3K/AKT pathways. Moreover, in their RNA-seq experiments, they highlighted the induction of *CDH10*, encoding for cadherin 10, upon MEOX2 depletion. However, in our RNA-seq results, we did not find evidence of modulation of *CDH10*, which remained stable in our GSCs transduced with anti-MEOX2 shRNAs. While we cannot exclude those technical differences (e.g., siRNA transient transfection vs. stable lentivirally-mediated shRNA expression, with the consequentially different levels of reduction in MEOX2 expression, possibly different in different transiently transduced cells, the timing of the experiments) can account for such divergent results, the different nature of the GSC lines used might be important to explain them too. Indeed, in the original subtyping classification of glioblastomas [4], MEOX2 was initially reported as a marker of the CL subtype, and more recently, MEOX2 was shown to be aberrantly activated and one of the master transcription factors in Group 1 GSCs [27]. Thus, except for technical issues, the difference between our results and those achieved by Tachon and collaborators might once again indicate a cell type-specific role of MEOX2.

Nevertheless, our results and those recently published in ref. n. 28 agree with the definition of MEOX2 as a key factor for different aspects of GSC biology. Further investigation and validation on a greater number of GSC lines are required to comprehend the molecular basis of MEOX2 action in depth and to overcome the preliminary nature of these functional studies, in turn, due to the limited number of GSC lines analyzed in both.

## 5. Conclusions

In conclusion, our findings support the role of MEOX2 as an important transcription factor in glioblastoma stem cells, where its depletion profoundly represses key genes of the glycolytic pathway involved in the Warburg effect along with several other genes engaged in the high ability of GSCs to respond to hypoxic and other types of stress, making them resistant to therapies and to the microenvironment where they reside.

## Figures and Tables

**Table 1:** Percentage of Annexin V (ANX), 7-amino actinomycin D (7-AAD), ANX/7AAD)-positive, and percentage of Hypodiploid nuclei in BT273 or BT379 GSCs transduced with shRNA18 or shRNA53 and analysed by Flow Cytometry.

Cells	Percentage (%)	ANX+7AAD-	ANX+7AAD+	ANX-7AAD+	Hypodiploid Nuclei
BT273	CTR	41.92 ± 12,71	7.62 ± 10.58	2.67 ± 2.07	10.19 ± 2.76
	shRNA18	* 62.19 ± 15.65	4.29 ± 4.14	3.40 ± 4.52	13.18 ± 0.05
	shRNA53	^+^** 71.79 ± 16.40	3.26 ± 1.01	7.79 ± 9.46	* 16.55 ± 1.04
BT379	CTR	29.37 ± 2.25	5.35 ± 3.34	7.05 ± 4.26	10.52 ± 6.61
	shRNA18	* 67.54 ± 5.25	6.46 ± 5.18	0.90 ± 0.37	** 43.52 ± 15.28
	shRNA53	^++^** 79.82 ± 3.38	12.99 ± 6.55	4.51 ± 4.58	** 47.46 ± 10.19

* *p* ≤ 0.01, ** *p*≤ 0.001 vs. CTR. ^+^ *p* ≤ 0.01, ^++^ *p* ≤ 0.001 shRNA 18 vs. shRNA53.

## Data Availability

All the data and material are available on reasonable request from the corresponding author.

## Supplementary Materials

The following supporting information can be downloaded at: https://www.mdpi.com/article/10.3390/cancers14092304/s1, Figure S1: MEOX2 expression characterizes glioblastoma stem cells; Figure S2: MEOX2 knock-down induces the repression of key factors of the glycolytic pathway; Figure S3: Enrichment analysis of transcription factor binding motifs in the regulatory regions of genes upregulated upon MEOX2 knock-down; Figure S4: Enrichment analysis of transcription factor binding motifs in the regulatory regions of genes downregulated upon MEOX2 knock-down; Table S1: Genes Differentially Expressed (DEGs) upon MEOX2 knock down; Table S2: Gene Ontology analysis of DEGs upon MEOX2 knock down; Table S3: oPOSSUM-3 analysis of regulatory regions of DEGs upon MEOX2 knock down; Table S4: oPOSSUM-3 analysis of regulatory regions of DEGs upon MEOX2 knock down, in each of the analyzed cell types (BT273 or BT379), transduced with either shRNA (shRNA18 or shRNA53), compared to the controls. File S1: Original Western Blot figures.
