# Supplementary material for: MEOX2 Regulates the Growth and Survival of Glioblastoma Stem Cells by Modulating Genes of the Glycolytic Pathway and Response to Hypoxia
Source: Cancers (Basel). 2022 May 6;14(9):2304. doi: 10.3390/cancers14092304 (PMC9099809; doi:10.3390/cancers14092304)
Supplement: Supplementary file 1 [file cancers-14-02304-s001.zip › cancers-1596268-figure S1-S4.pdf]

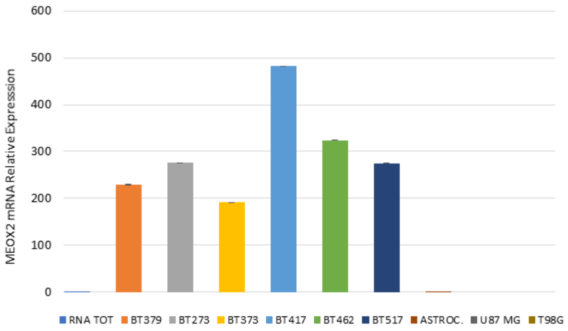

Figure S1. MEOX2 expression characterizes glioblastoma stem cells.
q-RT-PCR measurement of MEOX2 mRNA expression in total RNA extracted from healthy brain (RNA TOT), cultured healthy human astrocytes (astroc.), two established glioblastoma cell lines (U87MG and T98G), and six different human glioblastoma stem cell lines. The values were reported in relation to those found in the normal brain, set as = 1, and normalized to PPP2CA mRNA expression (n = 3; mean +/- SD).

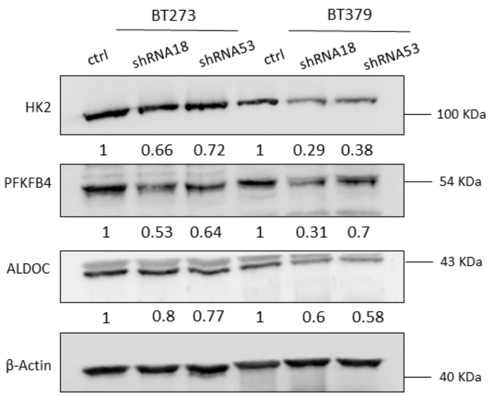

Figure S2. MEOX2 knock-down induces the repression of key factors of the glycolytic pathway.
Western blot analysis of HK2, PFKFB4, and ALDOC protein levels in BT273 and BT379 GSCs transduced with either the negative control or the shRNA18 or shRNA53 targeting MEOX2. Relative protein levels are indicated under each lane, and were quantified, in each cell line, in comparison to the cells transduced with the negative control vector, set as = 1. β-actin was used as the internal loading control.

Upregulated upon MEOX2 knock down

BT273 shRNA18

Z-score vs. TF profile %GC composition

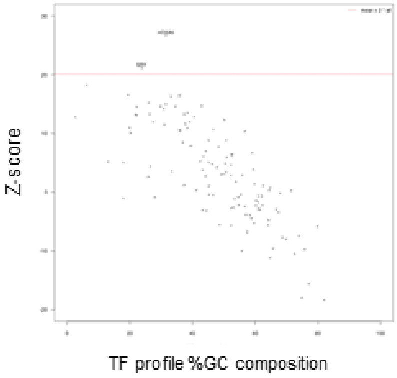

(a)

BT273 shRNA53

Z-score vs. TF profile %GC composition

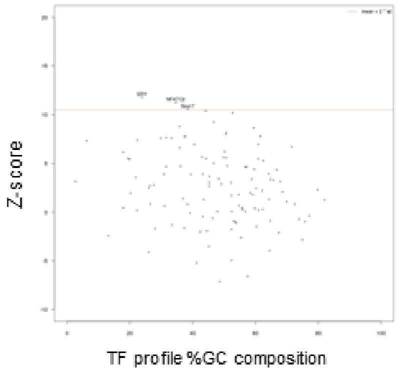

(b)

Figure S3. Enrichment analysis of transcription factor binding motifs in the regulatory regions of genes upregulated upon MEOX2 knock-down.
DEGs upregulated in BT273 transduced with shRNA18 (a), or shRNA53 (b), and in BT379 transduced with shRNA18 (c) were subjected to enrichment analysis of TF binding motifs using oPOSSUM-3 software. The names of the significantly enriched transcription factor binding motifs (Z score > mean + 2 SD) are shown.

BT379 shRNA18

Z-score vs. TF profile %GC composition

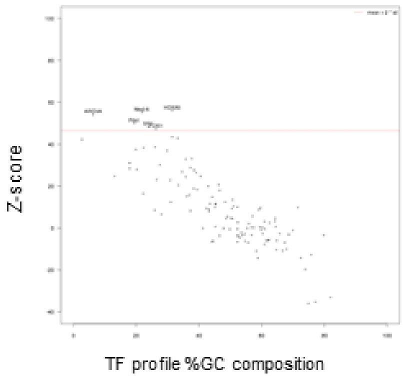

(c)

Downregulated upon MEOX2 knock down

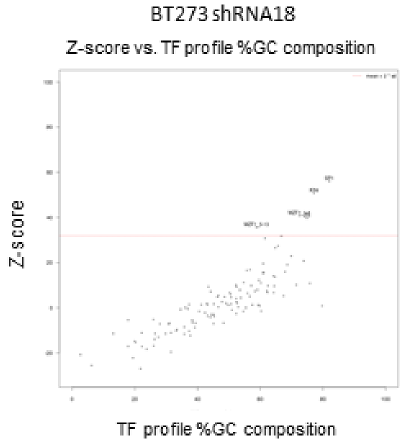

(a)

Figure S4. Enrichment analysis of transcription factor binding motifs in the regulatory regions of genes downregulated upon MEOX2 knock-down.
DEGs downregulated in BT273 transduced with shRNA18 (**a**), or shRNA53 (**b**), and in BT379 transduced with shRNA18 (**c**) were subjected to enrichment analysis of TF binding motifs using oPOSSUM-3 software. The names of the significantly enriched transcription factor binding motifs (Z score > mean + 2 SD) are shown.

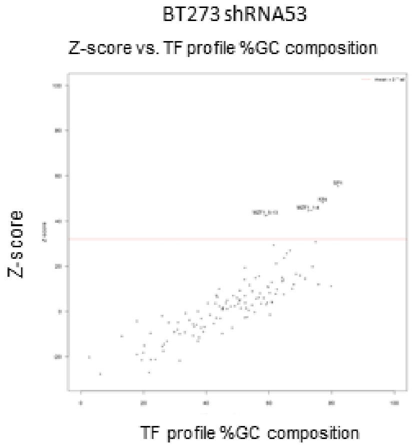

(b)

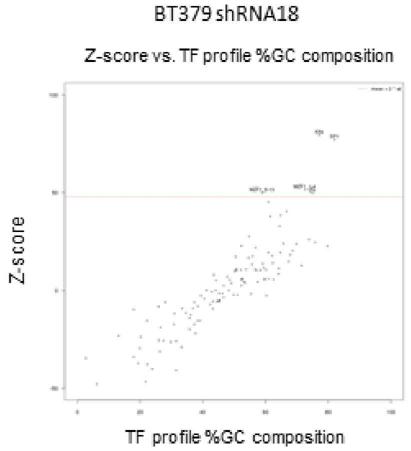

(c)
