# Supplementary figures and images for: MEOX2 Regulates the Growth and Survival of Glioblastoma Stem Cells by Modulating Genes of the Glycolytic Pathway and Response to Hypoxia
Source: Cancers (Basel). 2022 May 6;14(9):2304. doi: 10.3390/cancers14092304 (PMC9099809; doi:10.3390/cancers14092304)

Figure 1(c)

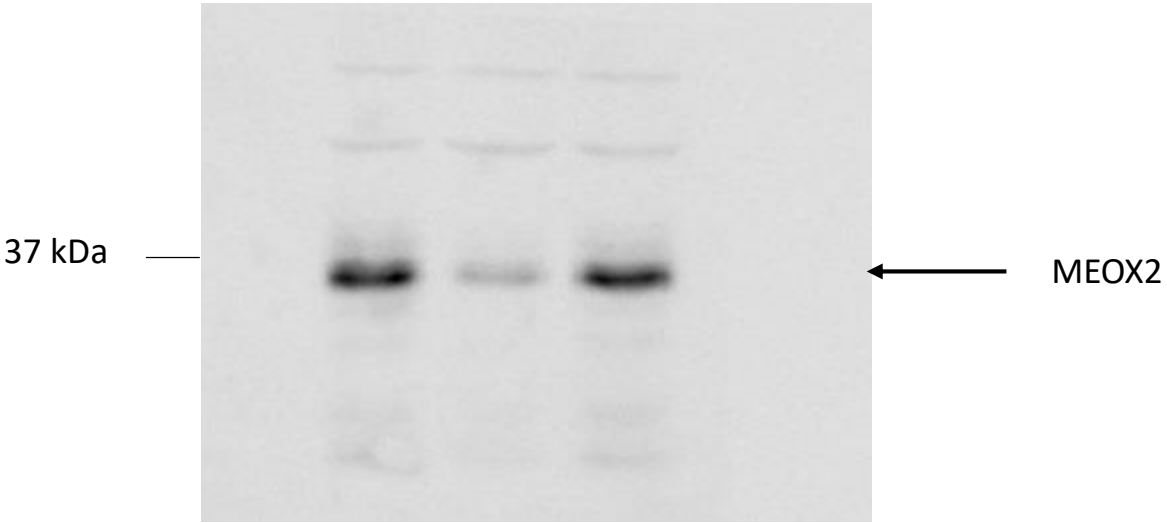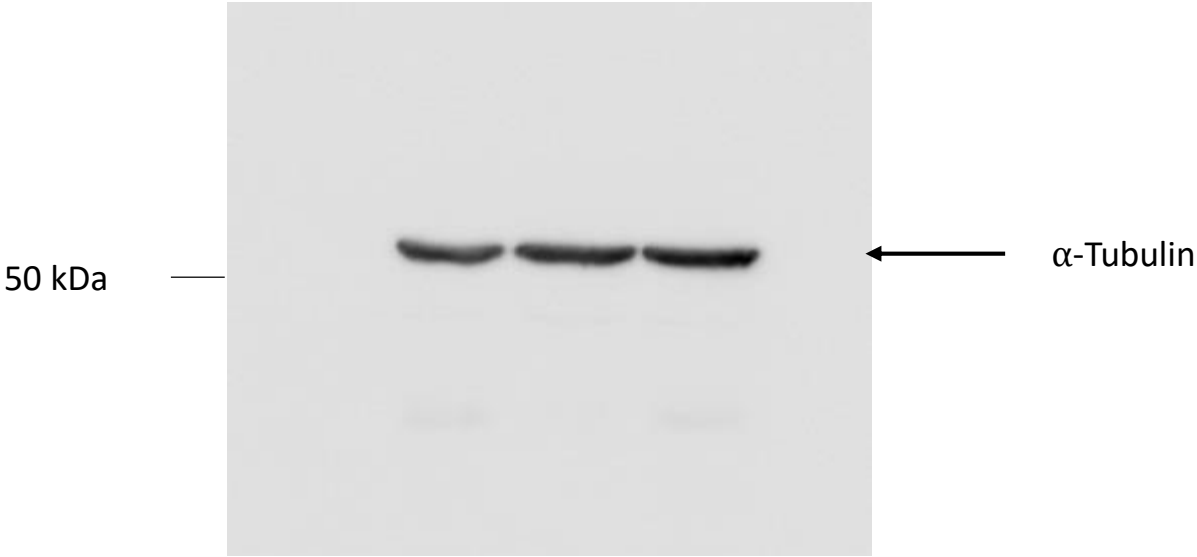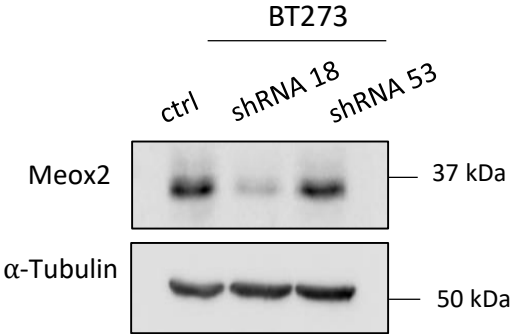

Figure 1(d)

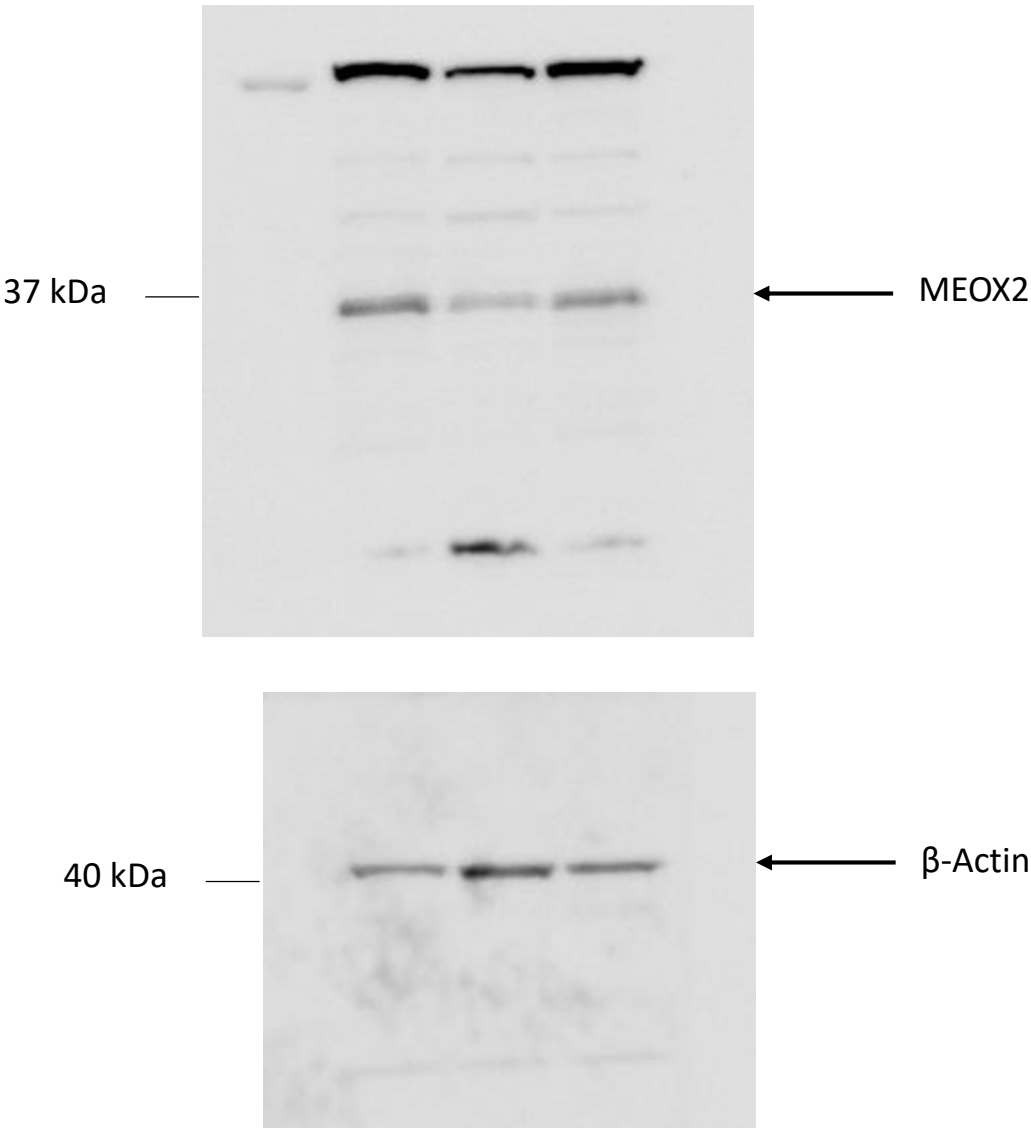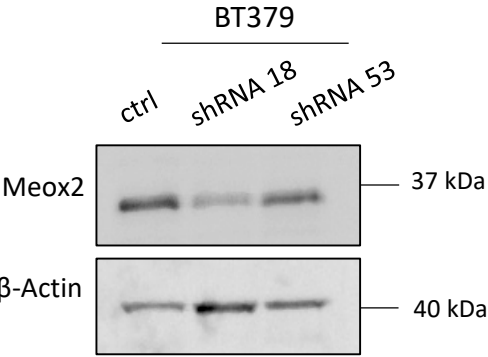

Figure 2(c)

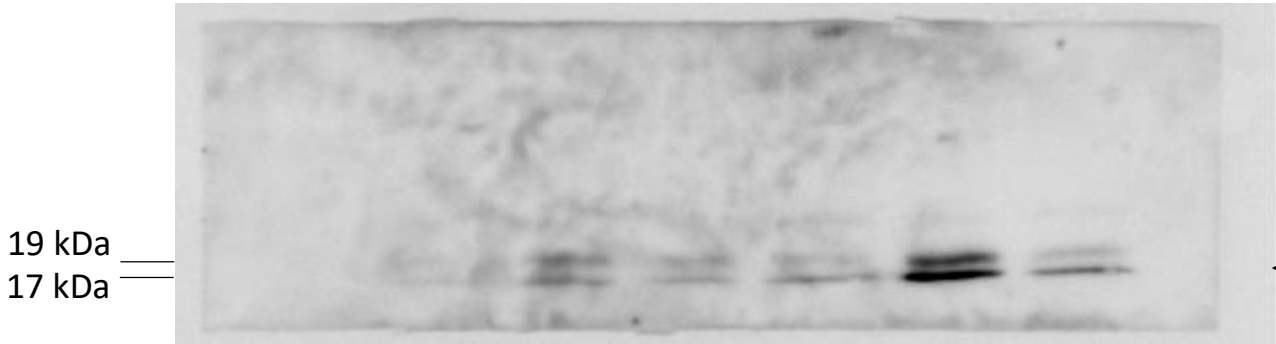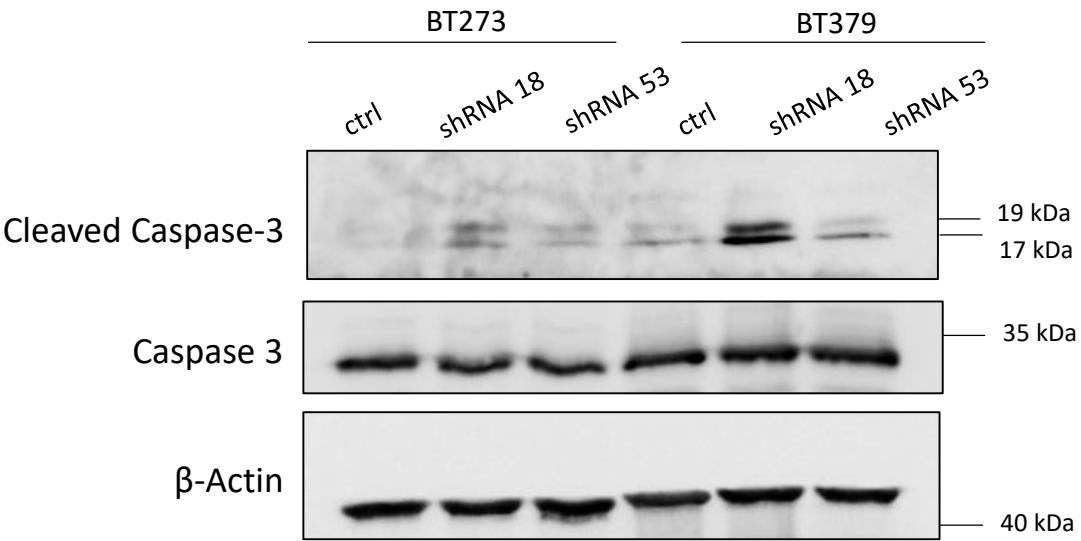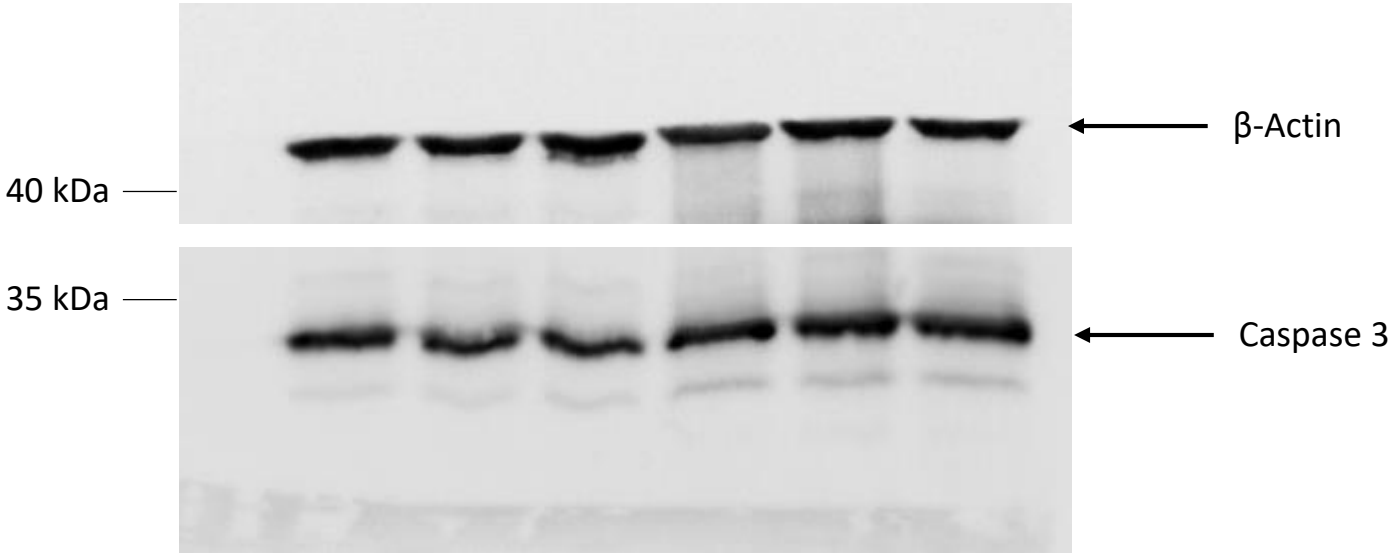

Figure S3

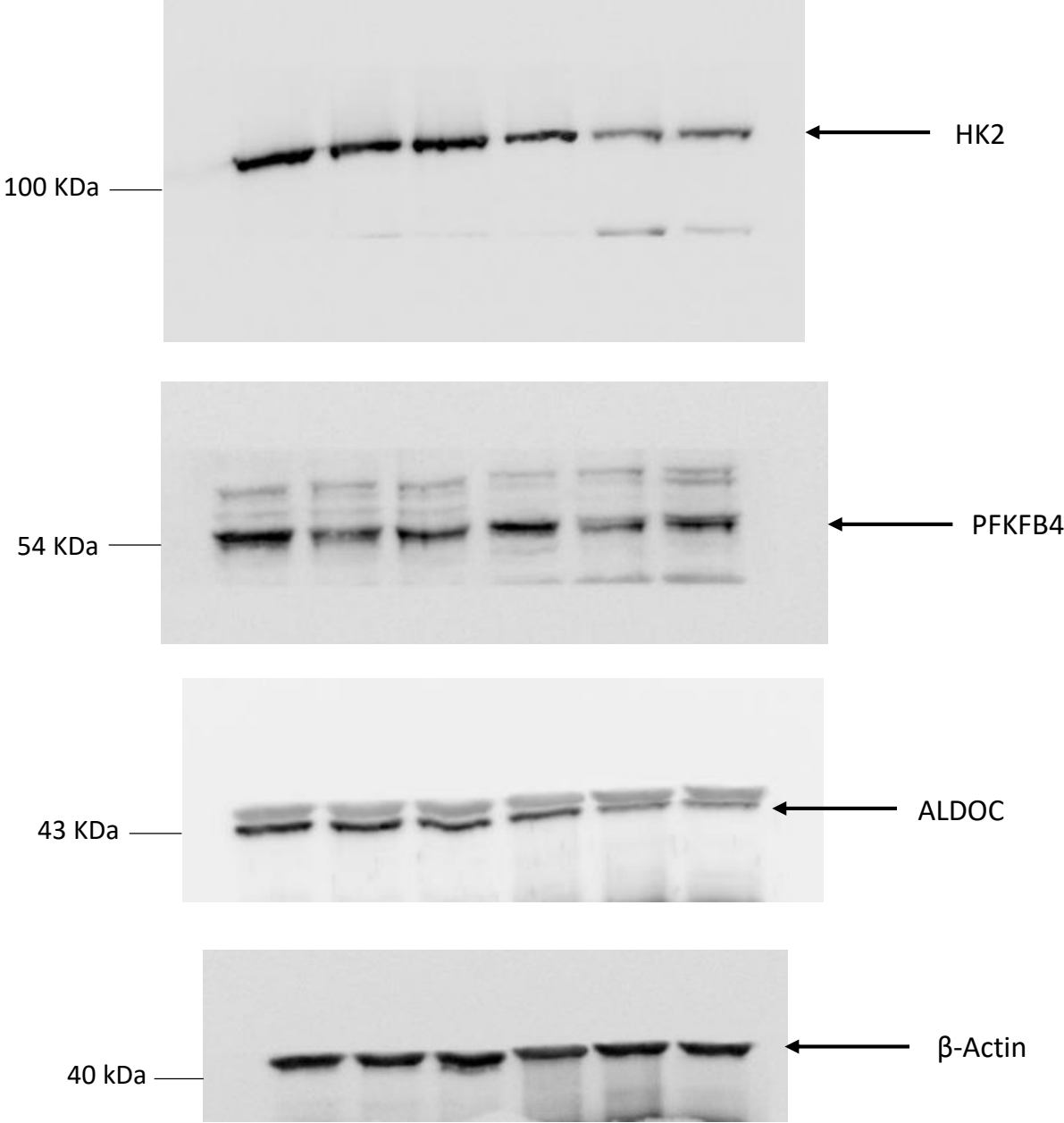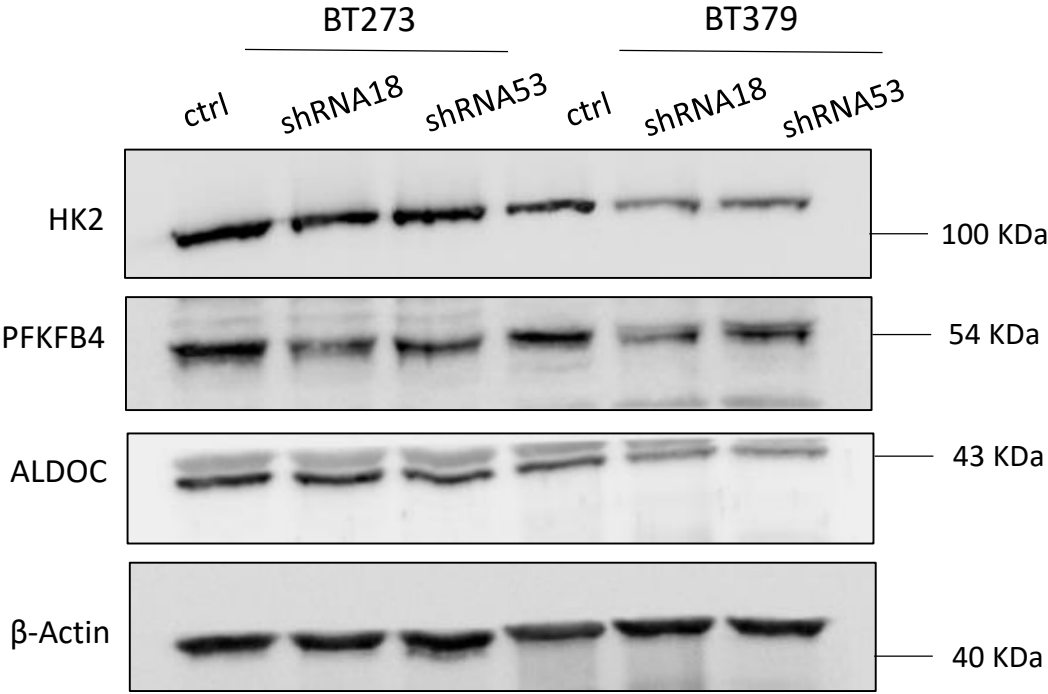

Supplement: Supplementary file 1 [file cancers-14-02304-s001.zip › cancers-1596268-file S1 - original image.pdf]
